# Genetic instability in the tumor microenvironment: a new look at an old neighbor

**DOI:** 10.1186/s12943-015-0409-y

**Published:** 2015-07-31

**Authors:** Antonio Palumbo, Nathalia de Oliveira Meireles Da Costa, Martin Hernan Bonamino, Luis Felipe Ribeiro Pinto, Luiz Eurico Nasciutti

**Affiliations:** Laboratório de Interações Celulares, Instituto de Ciências Biomédicas, Universidade Federal do Rio de Janeiro, Prédio de Ciências da Saúde - Cidade Universitária, Ilha do Fundão, A. Carlos Chagas, 373 - bloco F, sala 26, 21941-902 Rio de Janeiro, RJ Brasil; Programa de Carcinogênese Molecular, Instituto Nacional de Câncer José de Alencar Gomes da Silva, Rua André Cavalcanti, 37 - 6° andar - Centro, 20231-050 Rio de Janeiro, RJ Brasil; Fundação Oswaldo Cruz, Vice-presidência de Pesquisa e Laboratórios de Referência, Rio de Janeiro, Brasil, Av. Brasil, 4365 - Pavilhão Mourisco - Manguinhos, 21040-900 Rio de Janeiro, RJ Brasil

**Keywords:** Tumor microenvironment, Genetic alterations, Gene expression profile, Large scale analysis, Selective drugs

## Abstract

The recent exponential increase in our knowledge of cellular and molecular mechanisms involved in carcinogenesis has largely failed to translate into new therapies and clinical practices. This lack of success may result in part from the fact that most studies focus on tumor cells as potential therapeutic targets and neglect the complex microenvironment that undergoes profound changes during tumor development. Furthermore, an unfortunate association of factors such as tumor genetic complexity, overestimation of biomarker and drug potentials, as well as a poor understanding of tumor microenvironment in diagnosis and prognosis leads to the current levels of treatment failure regarding a vast majority of cancer types. A growing body of evidence points to the importance of the functional diversity of immune and structural cells during tumor development. In this sense, the lack of technologies that would allow for molecular screening of individual stromal cell types poses a major challenge for the development of therapies targeting the tumor microenvironment. Progress in microenvironment genetic studies represents a formidable opportunity for the development of new selective drugs because stromal cells have lower mutation rates than malignant cells, and should prove to be good targets for therapy.

## Introduction

The incidence of cancer has increased worldwide over the last century. In 2012, 32 million people had cancer within 5 years of diagnosis, 14 million new cases were diagnosed, and 8 million cancer deaths occurred worldwide [1]. According to the World Health Organization (WHO), within the next 15 years, over 17 million people will develop cancer annually, and in 2030, cancer will represent the leading cause of death worldwide, surpassing cardiovascular diseases [2]. Thus, cancer not only afflicts individuals, but it has become a social problem that burdens the public health systems which provide treatment and support to patients.

During the past decades, an increasing body of work has shed light on the cellular and molecular mechanisms involved in the transformation of normal cells into cancerous cells. However, this research largely fails to improve current clinical practices [3]. Physicians still treat a vast majority of tumors using relatively old protocols involving surgery, hormonal therapy, radio and chemotherapy [4]. New strategies including monoclonal antibodies and tyrosine kinase inhibitors explore molecular targets that are normally deregulated in cancer cells, and represent a new hope for patients. However, these therapies have only succeeded against a few types of cancers. Carcinomas, for instance, often fail to respond to molecular target therapies [5, 6]. Unresponsiveness may result, at least in part, because these therapies only target cancer cells and neglect the complex microenvironment around tumors that undergo drastic changes during disease development [7]. Furthermore, neglecting these changes in microenvironment cells could be a major oversight since, lately, it has been shown that the alterations found in stromal cells play a key role in both the progression and the initiation of several tumors [8, 9]. In this way, tumor microenvironment cells could represent an extremely attractive therapeutic target either along the course of the disease or during the first steps of malignant transformation.

### Reciprocal cellular interactions

Cellular interactions lie at the core of metazoan physiology and encompass an effective and elegant signaling repertoire that integrates up to trillions of cells in a single organism. These interactions begin during the early stages of embryonic development and coordinate events such as cell proliferation and differentiation in complex tissues, crucial processes for the functional maintenance of whole organs [10]. During adult life, tissue homeostasis is supported by direct cellular interactions and by the continuous exchange of soluble and non-soluble factors released by different cellular compartments [11].

Cell-cell interactions primarily occur between the mesoderm, endoderm and ectoderm during embryonic development and between the parenchyma and stromal compartments throughout adult life [12, 13]. In a vast majority of organs, distinct epithelial tissues form the parenchyma, whereas the stroma constitutes a complex compartment composed of different cell types including fibroblasts and myofibroblasts, vessel cells, pericytes, endothelial and smooth muscle cells, as well as immune cells such as macrophages, lymphocytes and mast cells. Finally, these different cell types are immersed in a complex protein network named extracellular matrix (ECM) [14].

### Cellular interactions in the tumor microenvironment

Carcinomas, which by definition arise from epithelial cells, account for nearly 80 % of all human cancers [1]. Epithelial cell transformation disturbs tissue homeostasis, but also causes extensive changes to the microenvironment surrounding the developing tumor [15–17]. Thus, the imbalance in homeostasis induced by cancer cells also promotes aberrant cellular behavior in the stroma, culminating in the complete alteration of reciprocal interactions mediated by this compartment [18]. This altered microenvironment, also known as reactive stroma, is histopathologically characterized by extensive phenotypic modifications, such as ECM remodeling, loss of smooth muscle cells, and persistent infiltration by myofibroblasts, as observed in a prostate cancer model [19].

Reactive stroma has been shown to emerge at the beginning of the disruption of the interactions with the epithelial compartment along prostate malignant transformation [20] and seems to be crucial to support the early steps of tumor progression in several tissues such as breast, ovary and liver [20–23]. A pivotal role in the generation of the reactive stroma is played by the Transforming Growth Factor β (TGF-β), which triggers the cell phenotype changes that characterize this compartment [20].

The TGF-ß superfamily is composed by different growth factors which play important roles in both physiological and pathological processes and, lately, has been associated with cancer development [24]. TGF- ß has dual functions in the cell, acting as tumor suppressor and oncogene. Even though in normal development TGF-ß acts as a differentiation and anti-proliferation factor in distinct cell types [25], in cancer, it leads to loss of cellular growth inhibition, proliferation activity, metastasis, angiogenesis, invasion and migration, favoring epithelial-mesenchyme transition (EMT) [25–27]. Under TGF-β influence, the stromal tissue, which is originally composed of a large amount of smooth muscle cells and molecular characterized by the expression of muscular differentiation proteins such as desmin, calponin, as well as myosin heavy chain, becomes more fibrous, displaying increased number of myofibroblasts, loss of muscular differentiation markers expression, along with an augmentation in the presence of the fibrous marker α smooth muscle actin [28]. The stromal phenotype change also induces ECM remodeling, resulting in the release of new growth factors and ECM molecules, such as collagen I and III [29], tenascin C and versican [30, 31], besides matrix metalloproteinases (MMP) 2 and 9 [32]. In addition, TGF-β inhibition was demonstrated to decrease the formation of new blood vessels in xenograft prostate cancer model [33], as well as apoptosis rates of prostatic myofibroblasts [34]. Besides TGF-β ability to promote ECM remodeling and, consequently, the release of angiogenic factors such as vascular endothelial growth factor (VEGF) [35], its angiogenic role also occurs through the boosted expression and release of angiogenic promoters such as Connective-Tissue Growth Factor (CTGF) and Fibroblast Growth Factor 2 (FGF-2) [36, 37]. Moreover, TGF-β directly regulates the expression of these two growth factors during the healing process where they are found involved with proliferation and migration signaling stimulus sent to fibroblasts, epithelial and endothelial cells [38]. These data reinforce the perspective that the alterations observed in the reactive stroma consist in a reedition of the mechanisms usually involved in the healing process [39], nevertheless, instead of arising from a specific biological context, the molecules controlling this response are constantly produced by the altered reactive stroma, thus contributing to promote important cancer hallmarks such as angiogenesis [40], invasion [41], metastasis [42] and the EMT [43].

The EMT seems to represent a breakpoint in enabling malignant cells to invade other tissues and organs. In fact, the EMT is primarily a cellular biological program typically involved in various stages of embryonic morphogenesis and wound healing, however, the wide repertory of molecular tools employed during this process is also used as a subterfuge by transformed cells to acquire skills related with invasion, metastasis and resistance to apoptosis [44]. This process is characterized by the transition from an epithelial and weak migratory phenotype to an intense migratory phenotype related with cells from mesenchyme origin, such as fibroblast cells [45]. One of the main characteristics of EMT is the repression of E-cadherin expression, which occurs through its regulation by several transcription factors including Snail, Slug, Twist and Zinc Finger E-Box Binding Homeobox (Zeb) 1/2. These transcription factors are also associated with matrix metalloproteinases expression and release that ultimately leads to an increase in cell motility and resistance to apoptosis [44]. In fact, the adherents junctions destabilization by E-cadherin repression is frequently observed in most of carcinomas [46]. Given the importance of the epithelial apical junctional integrity mediated by E-cadherin, its repression in malignant cells represents a crucial event that culminates in loss of adhesion between these cells and in enabling the cellular escape from its original site, which speak for the initial steps of tumor progression [47]. Nonetheless, recent studies have shown that E-cadherin loss is not sufficient to direct the EMT process in a mammary carcinogenesis model, since the initial events of cytoskeleton organization were architecture despite its expression absence [48, 49]. On the other hand, while genes related with growth inhibition and cell proliferation are typically downregulated in EMT, genes coding adhesion proteins normally associated with cellular migration during the embryogenesis and inflammation are found upregulated during this process [50], particularly N-cadherin and genes belonging to immunoglobulin superfamily [50, 51].

In this complex scenario represented by cellular interactions between stromal and epithelial cells, Weinberg and colleagues elegantly clarified these interplay during disease progression in two reports. In a study using *in vitro* and *in vivo* approaches, these authors showed that transformation of human mammary epithelial cells mediated by transfection with HRAS and hTERT increased cell proliferation and survival *in vitro*. Nude mice inoculated with these cells and mammary fibroblasts grew tumors twice as fast as mice inoculated with transformed cells alone [52]. This seminal work represented one of the first to show that genetic transformation *per se* does not confer all of the malignant characteristics observed in tumor cells, in particular those related with cellular invasion [53], epithelial-mesenchyme transition [54] and metastasis [55, 56]. In a subsequent study, the same group reported that fibroblasts derived from mammary tumors promoted malignant growth by locally inducing angiogenesis through the secretion of Stromal Derived Factor 1 (SDF-1), which attracts endothelial progenitor cells from the bone marrow [57]. However, in the same study it was noted that tumor growth was also supported in the presence of artificial ECM matrigel, even in the absence of fibroblasts. In fact, this could be partially explained by the well established role of ECM as a growth and angiogenic factors reservoir [58]. Furthermore, it had been previously demonstrated that ECM was able to modulate the expression of genes involved with malignant phenotype acquisition [16].

Since then, many studies have demonstrated the importance of the microenvironment in tumor progression. Recently, Maxwell and colleagues demonstrated that the release of the chemokine (C-X-C motif) ligand (CXCL)-8 in *PTEN* (phosphatase and tensin homolog deleted on chromosome 10) depleted prostate cancer cells up regulated the expression of chemokine receptors chemokine (C-X-C motif) receptors (CXCR) 1, 2 and 4 in the stromal compartment, besides augmenting the release of CXCL-12 and chemokine (C-C motif) ligand (CCL)2 that, in turn, by a paracrine signaling, sustained the aggressive behavior exhibited by malignant prostate cells [59]. The tumor suppressor gene *PTEN* controls cell survival and proliferation through the inhibition of PI3K/Akt intracellular signaling pathway [60]. *PTEN* is constitutively expressed in cells and figures as one of the genes most frequently mutated in several distinct tumors, demonstrating its importance in physiological processes [61]. The study published by Maxwell and colleagues shows that altered inflammatory chemokines release mediated by the loss of an important tumor suppressor as *PTEN*, represent an important axis in the interaction between stromal and malignant cells [62, 63].

In fact, the link between inflammation, cancer development and progression, first proposed by Virchow, is now a widely recognized process [64] currently described in details for several different tumors, including carcinomas of different origins [65–67]. Inflammatory components found in the tumor microenvironment such as macrophages, neutrophils, basophils, lymphocytes and other cell subsets establish interactions and crosstalk within the leukocyte compartment and with tumor cells orchestrating tumor progression and invasiveness.

The simple presence of leukocytes in the tumor mass does not allow discriminating the role these cells are playing in the tumor microenvironment. Leucocyte subsets can display different functions (CD4 T helper 1, 2, 17, regulatory T cells, CD8 cytotoxic T cells, regulatory B cells, immunosupressive myeloid derived suppressor cells –MDSCs- and others). Macrophages, for instance, display a full spectrum of functions that allow them to perform different tasks in the tumor microenvironment. These cells have been classically classified as M1 or M2 [68]. Macrophages classified as M1 are associated to cytotoxic activity on tumor cells and promotion of antitumor responses related to tumor vessels. These cells are related to type I interferon responses and reactive oxygen species burst. On the other hand, M2 macrophages are related to scavenger functions, IL-10 and arginase production, amongst other characteristics [69]. Macrophage polarization is closely related to functional polarization of other cell subsets, such as helper lymphocytes (Ths). Classic M1 cells are associated to Th1 (IFNg secreting) cells, while M2 cells correlate to Th2 (IL-4 and Il-13 secreting) cells, indicating a more complex and coordinated program that orchestrates more than one leukocyte subpopulation. Linked to IFNg producing Th1 cells, CD8+ effector lymphocytes can eliminate tumor cells and help to establish an anti-tumor microenvironment.

Other immune subpopulations have been described as playing roles in tumor progression, such as neutrophils (displaying the N1 and N2 polarization in cancer [70], recapitulating macrophages M1 and M2 status) and B lymphocytes [71, 72]. For some epithelial tumors, members of the innate and adaptive immunity seem to act coordinately promoting tumor progression. These networks include circuits described in mouse models recapitulating progressive spontaneous tumors. As examples, some recent descriptions include the mammary tumor cell-M2 macrophage and Th2 lymphocytes interplay in the MMTV-PyMT transgenic mouse breast cancer model [73] or the tumor cell - B lymphocyte activation and myeloid cell activation loop based on immunoglobulins and FcR interactions fostering squamous cell carcinoma progression in the K14-HPV16 mouse model [71].

Tumor cells not only recruit leukocytes to the tumor mass through chemokines (such as CSF-1) but are also stimulated by growth factors produced by the leukocytes (such as EGF produced by macrophages [74] and also modulate the function of these infiltrating tumor cells through the production of several immune modulators such as indoleamine 2,3-dioxygenase (IDO), TGF-β, Interleukin (IL)-10, arginase [75] or even lactate, as a result of the Warburg effect on tumor cell metabolism [76].

Taking into account the interplay of different leukocyte populations in the tumor, some groups are simplifying the evaluation of the complexity of tumor microenvironment by assuming correlations such as the ratio of CD4/CD8/CD68 (macrophage marker) in human tumors to estimate Th1/cytotoxic based responses and macrophages presumed to be M1 (if CD8/CD68 ratio is high), or M2 (if the ratio is low) [73].

Other groups take into account not only the function of the cells in the tumor microenvironment, but also different cell localizations, considering the relevance of the capacity of the cells to invade the tumor mass or to stay at the tumor margin [77, 78]. This approach has been named the Immunoscore, and represents a multi-center effort to demonstrate the predictive value of immune architecture evaluation in tumor samples. This approach is under extensive validation for some tumors such as colon adenocarcinomas [79] and highlights the relevance of characterizing the immune functions in the tumor biology field of study.

The immune architecture is not only a function of the immune cells recruited to the tumor site, but also of the intrinsic characteristics of tumor cells. As already mentioned, signaling loops have been extensively described between the tumor cells and immune system cellular components [66, 67, 71]. Nonetheless the activation status of components of the immune system have been associated with genotoxicity to tumor cells due to local production of reactive oxygen and nitrogen species [80] or the activation of mutation inducing machineries such as Recombination Activating Gene (RAG), Activation-induced Cytidine Deaminase (AID) [81] or Apolipoprotein B mRNA Editing Enzyme, Catalytic Polypeptide-Like (APOBEC) proteins [82].

In addition, it has already been reported that blood and lymph vessels network development also represents a notorious hallmark during carcinogenesis and tumor progression [44]. The peculiar nature of tumor vasculature not only provides the required conditions to malignant cells survival, spread and metastasize, but also creates a protective niche to tumor cells during disease development and treatment [83]. Inefficient lymph vessels drainage along with immaturity of tumor vessels creates a heterogeneous scenario mainly represented by hypoxia areas and variations in the interstitial pressure [83], that ultimately impacts in tumor progression and resistance to treatment [84, 85]. In this way, as key players in vessels development, the endothelial cells posses a prominent role in the disease [86]. In fact, the role of endothelium is not restricted to angiogenesis and vasculogenesis. Recent studies have reported that E-selectin expression in addition to EGF and IL-6 release by endothelial cells respectively mediates metastasis, EMT and cancer stem cells expansion [87–89].

Finally, the microenvironment has been implicated not only in tumor progression but also in carcinogenesis. Some clues initially originated at the end of the 1990s specifically associated MMP-3 expression and release with the development of breast cancer [90, 91]. Further, the authors dissected the mechanisms involved in the phenomenon by showing that MMP-3, which is normally overexpressed in breast tumor stroma [92], was capable of mediating the malignant epithelial transformation by inducing Rac1b expression. The increment of Rac1b expression increases ROS level that, in turn, produces DNA damage and genomic instability, besides promoting EMT activation by stimulating the expression of the transcription factor Snail [93]. Furthermore, additional researches have reinforced this perspective by showing that the contribution of the microenvironment is not an stealthy found, but a solid paradigm change in the carcinogenesis field [94–96].

### Genetic alterations in the tumor microenvironment

Since genes were identified as key elements in the carcinogenic process, they have become the focus of many cancer research projects. These studies have contributed to our knowledge of genetic alterations involved in disease generation and progression, including polymorphisms, mutations, translocations, recombination, and the regulation of gene expression. Moreover, this body of work has allowed for the development of tools currently used in disease diagnosis, prognosis and treatment [97]. The recent advent of large scale analysis techniques enabled researchers to investigate not only small sets of genes, but entire eukaryotic cell genomes. Such studies have contributed to the mapping of hundreds of genes associated with signaling pathways involved in cancer [98]. Large scale analyses, such as gene expression microarrays, have elucidated the gene expression signatures of many types of cancer, mostly carcinomas [99].

Unveiling the molecular constitution of tumors also revealed major differences in gene expression patterns of stroma surrounding normal and cancer tissues. For example, breast carcinoma associated fibroblasts (CAF), when compared to normal breast tissue fibroblasts, alter the expression of genes, in particular those associated with the healing process, such as PLAUR, LOXL2, PLOD2 and SDFR1 [100]. Analyses of solitary fibrous tumor (SFT) compared to desmoid type fibromatosis (DTF) revealed significant differences in stromal molecular profiles. The STF expression pattern correlated with decreased survival rates as a consequence of enriched expression of genes related with ECM remodeling, such as Collagen Type I and Type III and MMPs 11, 9 and 23; proliferation genes including Fibroblast Growth Factor (FGF), WNT5A, FZD 1 and 2; as well as angiogenic promoters such as TGF-β and CTGF [101]. On the other hand, DTF stromal gene expression correlated with better prognosis and overall survival not only for breast cancer, but also for ovarian, lung and colon tumors [102]. *In vitro* studies provided further evidence of the consequences of altered genetic expression in breast stromal cells: in co-cultures, up-regulation of CCL18 and CCL2 in CAF promoted breast tumor progression by inducing cellular invasion [103].

**Fig. 1:**
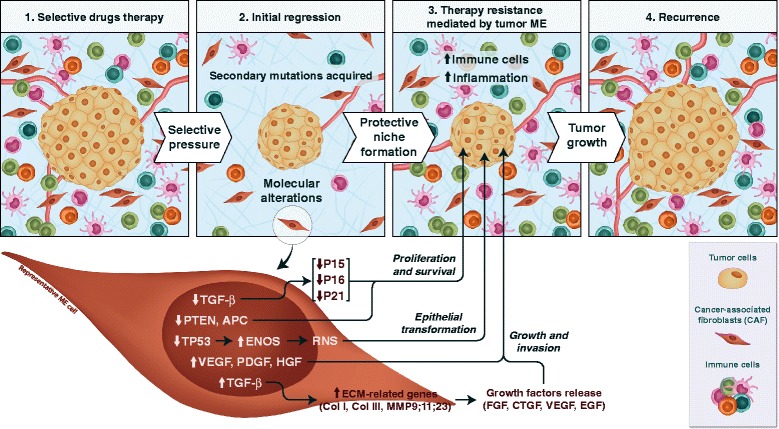
Molecular alterations found in the tumor microenvironment (ME) and the role of stromal cells in therapy resistance and disease recurrence. Upper panel: different steps comprised between initial therapeutic approach and tumor recurrence. **1** Tumor and ME cells at the beginning of selective drug therapy. **2** The selective pressure imposed by treatment contributes to the acquisition of secondary tumor mutations and genetic alterations in ME cells, which in turn affect tumor cells. ME alterations are represented in the CAF cell shown in the lower panel. Most of these alterations are associated with the loss of expression of tumor suppressor genes, such as PTEN and APC, which leads to increased tumor survival and proliferation. Furthermore, the loss of *TP53* expression observed in CAF increases ENOS activity, which culminates in an elevation of reactive nitrogen species (RNS) and the consequent transformation of the epithelial tissue surrounding the tumor. Moreover, the higher expression of growth factors (e.g., VEGF, PDGF and HGF) contributes to tumor growth and invasion, while TGF-β down-regulation decreases the expression of CDK inhibitors (p15, p16 and p21) in tumor cells, leading to tumor proliferation and survival. On the other hand, TGF-β up-regulation increases the expression of genes related with extracellular matrix (ECM) remodeling (Col I, Col III, MMP 9, MMP 11 and MMP23) which directly contribute to tumor progression. **3** ME cellular alterations, in addition to the recruitment of immune cells, and the increase in ME inflammation create a protective niche that results in therapy resistance. **4** The final outcome: tumor re-growth and disease recurrence

Large scale gene expression analyses performed in prostate tumors demonstrated that approximately 500 genes were up-regulated and 600 genes were down-regulated in tumor stroma [104]. Most differentially expressed genes participate in cancer-associated pathways, including the apoptosis pathway genes FOXL2, STAT1 and PPARγ; cell proliferation genes NOTCH1 and C-KIT; and DNA repair genes MRE11A, HUS1 and RAD17. Moreover, the same tumor stroma overexpressed Epidermal Growth Factor (EGF), FGF, TGF-β, Wnt and ECM related genes (Fig. 1), all of which participate in processes that ultimately disrupt interactions mediated by stroma during prostate cancer progression [105–107].

More recent work utilized the same approach to identify molecular alterations during the progression of esophageal adenocarcinoma. The authors reported that stromal samples from Barrett’s esophagus, a clinical condition caused by the long-term gastroesophageal reflux disease (GERD) that normally precedes the esophageal adenocarcinoma development, exhibits a peculiar inflammatory gene expression pattern, mainly represented by the TGF-β pathway. On the other hand, adenocarcinoma stromal samples overexpressed genes associated with poor prognosis including TMEPA, Thrombospondin 1, and BCL6 [108]. In this way, the authors conclude that esophageal stromal compartment presents a distinct gene expression signature, along the pathological steps involved in the disease progression.

In functional studies conducted *in vitro* comparing fibroblasts from normal human lungs and from non-small cell lung-cancer, the latter overexpressed genes involved in the TGF-β and MAPK signaling pathways. These findings were corroborated by parallel ex-vivo analyses of fresh tissue samples, and activation of the signaling pathways correlated with a worse prognosis [109]. Ovarian [110] and colon carcinomas [111] also display extensive stromal alterations. In addition to the effects of broad gene expression changes in the tumor microenvironment, a critical point for disease progression resides in the status of the master gene Tumor Protein p53 (*TP53*). Many studies have extensively explored the roles of *TP53* in tumor cells [112–114]. More recent work, however, has focused on the loss of functional *TP53* in the stroma of different tumor types [115–119].

In this way, data produced by Hill and colleagues showed that conditional deletion of *Rb* gene in mice epithelial cells not only allows tumor formation by prostate cells, but also leads to the loss of *TP53* in the stromal compartment, abrogating the homeostasis between epithelial and stromal tissues and ending up with loss of *TP53* also in the tumor [118]. These observations raised the hypothesis that the well reported loss of *TP53* in most of human carcinomas could be preceded by *TP53* loss in stromal cells which, in turn, would happen as a consequence of an initial driver genetic event in epithelial tissue, such as *Rb* gene loss, responsible for not only promoting a permissive microenvironment propitious to initiated cells, but also for contributing to an important event in tumor progression which is *TP53* loss. A provocative hypothesis conjectured by Hill and colleagues suggested that oncogenic stress mediated by initiated epithelium would create a selective pressure in the microenvironment that culminates with p53-deficient stromal cells selection [118]. Of note, this hypothesis was later partially corroborated by a study which showed that in breast and lung xenograft cancer models the number of malignant cells impacts on the oncogenic stress intensity that, in turn, induces the selection of p53deficient stromal cells [120]. The study of Farmaki and colleagues proposed that the large amounts of growth factors produced by malignant cells, such as EGF, creates a mitogenic stress that impairs the proliferation of p53-wild type fibroblasts, nevertheless, this stimulus is ineffective to p53-deficient fibroblasts, that are consequently selected in the tumor microenvironment [120]. A crucial limitation that could compromise the interpretation of the data achieved in the above cited studies is the joint inoculation of p53-wild type and p53-deficient fibroblasts. In this way, the data produced by Farmaki and colleagues not efficiently clarified if the intrinsic selective advantage provided by *TP53* absence was achieved due to the selective pressure imposed by malignant cells through oncogenic stress or due to the presence of p53-deficient fibroblasts since the beginning of the process. Despite the fact that the precise mechanisms of *TP53* loss in stromal cells had not been elucidate, the oncogenic stimulus emerges as a pivotal player in this process, since classical molecular alterations in the epithelial compartment, such as k-Ras mutation, were reported as sufficient to inhibit *TP53* expression in the fibroblasts [121].

Alternatively, the consequence of *TP53* loss in stromal cells has been particularly implicated in tumor progression, once its absence is inversely proportional to SDF-1 cytokine levels that, in turn, are involved with cellular proliferation and migration stimulus [122]. In fact, it was previously demonstrated that *TP53* overexpression in fibroblasts leads to a down-regulation of *SDF-1* and consequently attenuate migration and invasiveness processes [123]. Moreover, as previously discussed, SDF-1 promotes angiogenesis by stimulating the recruitment of endothelial progenitor cells [57]. Furthermore, homeostasis disruption promoted by *TP53* loss in the microenvironment cells is also apparently involved with alterations in the cellular redox state. During *in vitro* assays, stable silencing of *TP53* in fibroblasts resulted in overexpression of Endothelial Nitric Oxide Synthase (eNOS) (Fig. 1). Subsequently, reactive nitrogen species accumulation produces an unbalanced redox state, a well-known phenomenon related with alterations in the expression and function of many proteins [124]. The redox unbalance produced by *TP53* loss in fibroblasts cells, was strikingly related with an increase in mRNA and protein expression on Cytokine Intercellular Adhesion Molecule 1 (ICAM1), as well as in its incremented release. Moreover, these alterations culminated in the transformation and invasion of epithelial cells from the ovaries and oral cavity [125].

In fact, oxidative stress damages DNA in tumor cells, but also in stromal cells, contributing to an even greater disruption of microenvironment homeostasis during tumor progression [126, 127]. Moreover, the importance of *TP53* status in microenvironment was reinforced by the observation that the melanoma cell line B16F1 inoculated in p53-null mice produces tumors greatly faster than p53-wild type mice [63]. Increased amount of myeloid derived suppressor cells and leukocytes together with decreased number of CD8 lymphocytes was observed in tumors originated in animals lacking *TP53*. This scenario propitiated tumor growth by promoting an immunotolerant and permissive microenvironment which was evidenced by IFN-γ- and IL-17A suppression. In addition, the exacerbated release of G-CSF, CXCL1 and IL-6 chemokines creates an inflammatory context that promotes malignant proliferation and cell spread by sustaining the angiogenesis process [63].

In addition to *TP53*, the loss of function of other tumor suppressor genes in tumor stroma has severe consequences. For example, studies report that loss of function of genes involved in cell survival and proliferation, such as the *APC* (Adenomatous Polyposis Coli) and *PTEN* genes (Fig. 1), strongly contributes to endometrial and breast cancer development, respectively. *APC* deletion was associated with the development of an advanced malignant phenotype represented by myofibroblast infiltration, release of angiogenic factor promoters, including VEGF and SDF, and a decrease in responsiveness to progesterone and estradiol via reduction of receptor expression [128]. *APC* gene is a tumor suppressor mostly known by negatively regulating the Wnt/ß-catenin pathway, nevertheless, it controls many other cellular functions such as migration, regulation of apical-basal polarity, microtubule networks, cell cycle, DNA replication and repair and apoptosis that are involved in the organization of epithelial tissues [126, 127, 129].

On the other hand, *PTEN* loss results in Ets2 overexpression and activation, which in turn promotes immune cell infiltration and angiogenesis, as well as ECM remodeling [130, 131]. Ets2 belongs to the Ets (E26 transformation-specific) transcription factor family, which comprises approximately 30 evolutionary conserved members that are frequently found deregulated in cancer [132–134]. Ets2 overexpression has already been demonstrated to stimulate cell proliferation and tumor progression [135, 136].

In other hand, as previously reported, TGF-β represents an important frequently altered gene in the tumor microenvironment. This growth factor acts as a general coordinator of microenvironment homeostasis in most types of tumors, [137] and its signaling pathway is normally activated in most tumor stromal compartments [101, 108]. On the contrary, the absence of TGF-β pathway activation also figures as a critical event in some situations, specifically when its signaling pathway disruption is mediated by the lack of Transforming Growth Factor Beta Receptor II (TGFBR2), as observed in colon and esophageal carcinomas [138, 139]. Particularly in esophageal squamous cell carcinoma, deletion of TGFBR2 in mouse fibroblasts *in vivo* resulted in extensive genetic and epigenetic alterations, such as loss of cyclin inhibitors p15 and p16, as well as hypermethylation of the p21WAF1 promoter in surrounding epithelial cells (Fig. 1) that ultimately developed into esophageal squamous cells carcinomas [139].

On the other hand, genetic lesions in the tumor cells have also been described to influence the characteristics of the leukocytes infiltrating the tumor mass. For instance, some genetic deletions comprising regions bearing leucocyte stimulating factors can be deleted leading to poor local lymphocyte proliferation, as shown for the IL-15 deletion in colorectal tumors [140]. In this case, the limited expansion of lymphocytes leads to impaired anti-tumor responses, assuring constant tumor growth. Genetic deletions of regions including other immune mediator have been described in some tumors, such as IFNg locus deletions in melanomas [141], although the relevance of these events in terms of tumor immune architecture is still to be determined.

Alternatively, genetic studies on tumor microenvironment have shown that, despite the important changes observed in gene expression in a wide range of tumors, [128, 131, 142–145] this compartment is normally genetically more stable than epithelial tissues, exhibiting low mutation rates [146, 147]. Taking advantage of a SNP array platform, Qiu and colleagues showed that CAFs derived from ovarian and breast cancer rarely exhibit alterations in gene copy number and LOH [146]. This observation launched the discussion on whether the majority of the genetic alterations mapped in tumor microenvironment could be a result of the limitations of the techniques employed in the diverse studies. In the same study, this question was elegantly raised by authors due to the observation that when a fraction of DNA derived from tumor epithelial cells was combined with normal DNA, all the major alterations previously mapped in the tumor sample were also detected in the mixed sample, suggesting that a simple tissue contamination would be sufficient to mask the results. In fact, some studies reported a high frequency of genetic alterations in tumor microenvironment cells. In some cases, the alteration load was comparable to that observed in epithelial cells, with LOH frequency near 60 % in CAFs derived from ovarian and breast cancer [143, 145], supporting the idea that sample contamination and technical limitations could account for the surprising results achieved. Tissue microdissection and *in vitro* cell segregation were presented as possible alternatives for the technical issues faced. Nevertheless, Alinen and colleagues published an interesting study in which was demonstrated that all breast cancer cell types exhibited changes in gene expression, however, genetic alterations were only detected in malignant cells [148].

Further, a similar study corroborated the results produced by Alinen and colleagues and reported that only one sample, out of 25 CAFs obtained from 25 fresh breast samples, exhibited a chromosomal aberration involving the chromosomes 4, 6 and 9 and another one a *TP53* point mutation [147]. In addition, the role played by epigenetic alterations in controlling the gene expression changes exhibited by microenvironment cells definitively cannot be excluded and emerges as a promising field to explain the deranged behavior of microenvironment cells, particularly in tumor progression [149].

Most studies focusing on microenvironment molecular characterization describe patterns involving the entire stromal compartment without discriminating alterations in specific cell types that compose this niche [150]. In part, this limitation may result from technical difficulties in efficiently separating different stromal cell types even *in vitro*. Isolating immune cells from human solid tumor samples, for example, is especially challenging [151]. Nevertheless, given the wide functional diversity exhibited by immune and structural cells during tumor progression [152–155], molecular screening of individual cell types in the microenvironment represents a challenge that cannot be ignored.

### Exploiting new targets

**Fig. 2:**
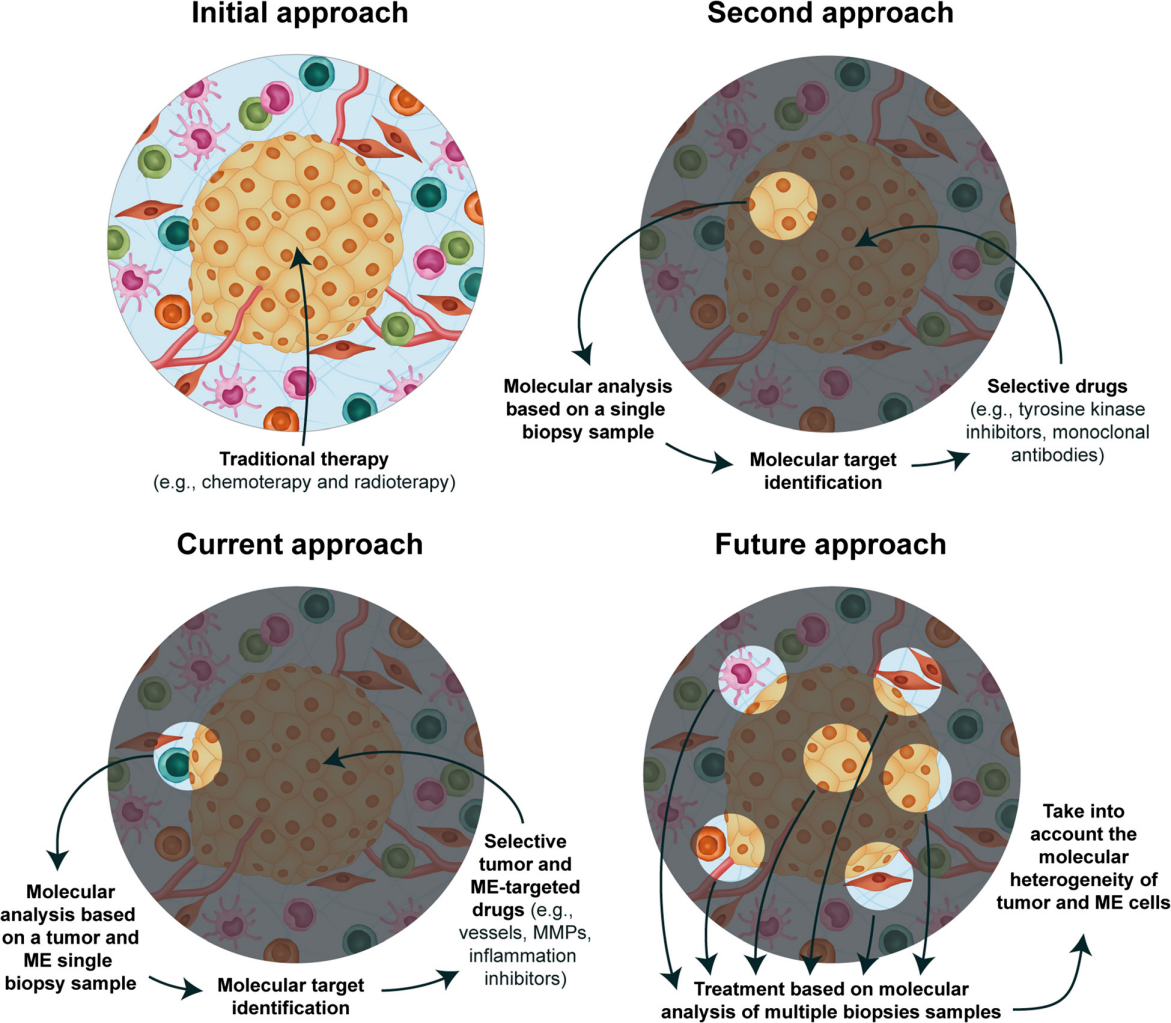
Evolution of cancer therapies over the years. The *initial approach* represents conventional therapy, which is based on the employment of unspecific chemical and physical agents (chemo and radiotherapy) that target general cellular processes occurring in both healthy and cancer cells and that do not take into account molecular alterations exhibited by the tumors. In the *second approach* **,** the molecular screening of cancer cells led to the development of selective drugs based on altered molecular features of malignant cells. This approach is particularly represented by tyrosine kinase inhibitors and monoclonal antibodies, which specifically target tumor cells. It is based on single-biopsy studies and does not take into account molecular alterations exhibited by microenvironment (ME) cells or tumor heterogeneity. The *current approach* shows that, lately, not only the tumor, but also its ME, have been mapped, which contributed to the development of selective drugs designed for both malignant and ME cells. The ME alterations more frequently targeted by molecular drugs are the ones observed in several types of cancers, such as angiogenesis, inflammatory processes and MMP overexpression. Again, this approach is based on single-biopsy studies and does not account for tumor heterogeneity. The *future approach* for cancer treatment shall account for the whole molecular heterogeneity, which is differentially exhibited by malignant and ME cells and unveiled by multiple biopsies

The development of cancer therapies started in the 19^th^ century. Since then, many physical, biological and particularly chemical agents have been used in cancer treatment [156]. As a result of tumor biology, most therapeutic agents focus on the interruption of malignant growth through the induction of cell death and/or proliferation arrest [157]. Traditional anti-carcinogenic agents differ in their precise mode of action, but in general, they either interfere with DNA or with important organelles involved in cytoskeleton integrity [69]. This overall pattern is represented by the leading approaches in cancer treatment: chemotherapy and radiotherapy. Since their development over 50 years ago, these therapies, alone or in combination, represent the principal line of disease control and increased survival [157, 158]. Nevertheless, lack of specificity poses a notorious problem: both healthy and cancer cells are targeted and the whole organism is heavily affected (Fig. 2). Furthermore, acquired resistance to chemotherapeutic agents and to specific target drugs represents a great challenge concerning cancer treatment [158].

The improvement of DNA-based technologies over the past decade brought great expectations about the discovery of new molecular targets for selective drugs (Fig. 2) [159, 160]. Nevertheless, these hopes were in great part frustrated by the unfortunate association of a range of factors such as overestimated drug potential and poor knowledge of tumor microenvironment [161, 162]. Therapeutic failure often relates to high mutation rates of tumor cells and the consequent intratumoral heterogeneity [163]. In a large number of cases, heterogeneity results from a peculiar nonlinear tumor evolution that stems from important master mutations and the development of multiple secondary mutations resulting from the pressure exerted by environmental factors such hypoxia or even the treatment itself (Fig. 1) [163, 164].

This complex scenario has elevated the failure rates of cancer treatments, particularly regarding selective drugs. These drugs should interact with specific targets that are frequently altered in malignant cells and do not represent the entire genetic mosaic of a tumor. This characteristic may partly result from the fact that some drugs are prescribed from single biopsy studies (Fig. 2) [165].

In other way, some patients with non-small cell lung cancer become refractory to Epidermal Growth Factor Receptor (EGFR) inhibitors due to an EGFR mutation or MET amplification that arises after treatment with Getifinib and Erlotinib [166, 167]. Similar results were observed in non-small cell lung cancer patients treated with the ALK inhibitor Crizotinib. These patients relapse after the onset of secondary alterations, such as ALK fusion gene amplification or increased EGF phosphorylation [168]. Nearly 60 % of melanoma patients harbor mutations in the *BRAF* gene. However, *BRAF* pathway inhibitors often fail as a result of secondary mutations to the Platelet-Derived Growth Factor Receptor (PDGFRB) and NRAS [169]. Moreover, despite the celebrated success of BCR/ABL fusion gene inhibition in chronic myeloid leukemia patients, continuous treatment occasionally produces drug resistance via acquisition of novel BCR/ABL mutations [170] or alternative mechanisms, and the leukemia stem cells seem to be resistant to BCR/ABL inhibitors [171]. Additionally, recent studies have reported that acquired resistance to some cancer therapies is not necessarily mediated by malignant cells but by tumor microenvironment [172]. In fact, provocative results published by Straussman and colleagues showed that therapy resistance exhibited by tumor cell lines that harbor the BRAF (V600E) mutation was mediated by receptor activation (MET) and stromal-cell derived Hepatocyte Growth Factor (HGF) [173]. Furthermore, it was reported that permanent secretion of proliferation factors such as WNT16B and the formation of a protective niche by stroma during treatment comprise one of the main limiting factors to successful cancer therapies (Fig. 1) [174, 175]. In addition to these findings, the role of immune cells in cancer has been established and their contribution to resistance to traditional and new therapies has been associated with cellular infiltration patterns [176, 177].

For instance, the presence and function of cells such as M2 macrophages can impact not only the natural progression of the tumor, but has also been demonstrated to impact the tumor response to chemo [73] and radiotherapy [178], outlining the relevance of characterizing the presence of these cell populations and ultimately of being able to manipulate the tumor leucocyte components to foster the efficacy of the treatment [179].

Presumably, these reports pushed forward the development of drugs that target the microenvironment (Fig. 2), such as metalloproteinase inhibitors (Tanomastat, Maromastat and Prinomastat), vessel inhibitors (Bevacizumab, Vandetanib, Sunitinib, Axtinib, Sorafenib, and others) and immune-cell modulators (Aldesleukin, Interferon Alpha 2b, Sipuleucel T, Ipilimumab, and others). Some of these therapies have displayed relative clinical success such as Vascular Endothelial Growth Factor Receptor (VEGFR)-targeted drugs [180, 181], whereas others, such as metalloproteinase inhibitors, have exhibited controversial or no results [182].

Immune therapies represent a more complex landscape due to the fact that the dual roles of the innate and adaptive immunological responses have yet to be completely understood [183]. Nevertheless, some encouraging results have been reported, especially when immune therapy is combined with traditional treatments or even with selective drugs [184, 185].

Tumors co-evolve with the immune response avoiding immune surveillance and ultimately leading to tumor progression and metastasis [186]. Tumor response to cytokines produced by leukocytes can lead to the activation of one of such escape mechanisms, leading to the impairment of immune response to tumors. This is the case for instance of the programed death ligand 1 (PDL1) up regulation on tumor cells upon local IFNg production by infiltrating lymphocytes [187]. The PD1 receptor of recently activated T lymphocytes is engaged by PDL1 on tumor cells leading to the impairment of T cell function. This process can be reverted by using monoclonal antibodies designed to perturb this interaction, unleashing lymphocytes to exert their antitumor effect [188], in a clear example of targeted therapy approaching natural immune mediated antitumor responses. Immune based antitumor responses can be also induced or boosted through vaccination approaches [189] or by simply expanding *in vitro* tumor specific lymphocytes to be returned to the patient, a strategy that leads to massive antitumor responses in a fraction of patients bearing metastatic melanomas [190]. A more refined derivation of this approach includes cloning and transgenically transferring T cell receptors to T lymphocytes [191] or chimeric antigen receptors (CARs) [192, 193] specific for tumor antigens. These strategies have proven able to induce tumor remissions in hematopoietic tumors, especially for CAR utilization in CD19+ target tumors [194] and will likely prove efficient also in solid tumors [192, 195]. The recent resultsobtained by several groups prove again the huge potential of understanding the basic mechanisms underlying the immune evasion by tumors and the best maneuvers to tackle these processes, leading to tumor elimination.

Research on the tumor microenvironment as a target for cancer therapy has only just begun when compared with research on tumor cell targets, and, therefore, the benefits of this approach are not yet clear. The future development of new drugs, which will take into account cancer cells and their microenvironment (Fig. 2), is needed and depends on a better understanding of stromal biology, anchored on morphological, phenotypic and genomic studies. Considering the concepts and ideas exposed in this review, it is reasonable to consider the microenvironment as a formidable opportunity for the development of new selective drugs since these cells provide an exciting potential target that may exhibit lower mutation rates than malignant cells.

## Conclusion

Genetic determinism has dominated cancer research and produced invaluable insights. However, the simplistic somatic mutation theory (SMT), which states that genetic instability of malignant cells alone drives disease progression, has proved insufficient to explain tumor behavior and, consequently, to point to new disease therapies. A recent, more dynamic view of tumors suggests that disease progression is promoted by the orchestrated interaction between malignant cells and their surrounding environment. Therefore, the development of more effective new therapies requires a global view that integrates the genocentric and microenvironmental knowledge in a plural approach.

## Abbreviation

AID: Activation-induced cytidine deaminase
APC: Adenomatous polyposis coli
APOBEC: Apolipoprotein B mRNA Editing Enzyme, Catalytic Polypeptide-Like
CAF: Cancer associated fibroblasts
CCL: Chemokine (C-C motif) ligand
CTGF: Connective-tissue growth factor
CXCL: Chemokine (C-X-C motif) ligand
CXCR: Chemokine (C-X-C motif) receptors
DTF: Desmoid type fibromatosis
ECM: Extracellular matrix
EGF: Epidermal growth factor
EGFR: Epidermal growth factor receptor
EMT: Epithelial mesenchyme transition
eNOS: Endothelial nitric oxide synthase
FGF: Fibroblast growth factor
HGF: Hepatocyte growth factor
IARC: International Agency for Research on Cancer
ICAM1: Intercellular adhesion molecule 1
IFN: Interferon
IL: Interleukin 10
MMP: Matrix metalloproteinase
PDGFRB: Platelet-derived growth factor receptor, beta polypeptide
PTEN: Phosphatase and tensin homolog
RAG: Recombination activating gene
SDF-1: Stromal derived factor 1
SFT: Solitary fibrous tumor
SMT: Somatic mutation theory
TGFBR2: Transforming growth factor, beta receptor II
TGF-β: Transforming growth factor β
Th: Helper lymphocyte
*TP53*: Tumor protein p53
VEGF: Vascular endothelial growth factor
VEGFR: Vascular endothelial growth factor receptor
WHO: World Health Organization
ZEB: Zinc finger E-Box binding homeobox
